# The Effect of Low Tidal Volume Ventilation on Inflammatory Cytokines During Cardiopulmonary Bypass

**DOI:** 10.21470/1678-9741-2020-0466

**Published:** 2022

**Authors:** Nazım Kankılıç, Mehmet Salih Aydın, Mustafa Göz

**Affiliations:** 1 Department of Cardiovascular Surgery, Medical School of Harran University, Şanlıurfa, Turkey.

**Keywords:** Cardiopulmonary Bypass, CPB (Incl Set-Ups, Equipment, Surface Coatings, Etc.), Inflammatory Mediators (Eg, Cytokines, Cytotoxins, Metalloproteinases), Complement, Lung Volume Reduction

## Abstract

**Introduction:**

Halting ventilation during cardiopulmonary bypass (CPB) is implemented to operate in a less bleeding setting. It sustains a better visualization of the operation area and helps to perform the operation much more comfortably. On the other hand, it may lead to a series of postoperative lung complications such as atelectasis and pleural effusion. In this study, we investigated the effects of low tidal volume ventilation on inflammatory cytokines during CPB.

**Methods:**

Twenty-eight patients undergoing cardiovascular surgery were included in the study. Operation standards and ventilation protocols were determined and patients were divided into two groups: patients ventilated with low tidal volume and non-ventilated patients. Plasma samples were taken from patients preoperatively, perioperatively from the coronary sinus and postoperatively after CPB. IL-6, IL-8, TNF-α and C5a levels in serum samples were studied with enzyme-linked immunosorbent assay (ELISA) kits.

**Results:**

C5a, IL-6, IL-8 and TNF-α were similar when compared to the low tidal volume ventilated and non-ventilated groups (*P*>0.05) Comparing the groups by variables, IL-6 levels were increased during CPB in both groups (*P*=0.021 and *P*=0.001), and IL-8 levels decreased in the ventilation group during CPB (*P*=0.018).

**Conclusion:**

Our findings suggest that low tidal volume ventilation may reduce the inflammatory response during CPB. Although the benefit of low tidal volume ventilation in CPB has been shown to decrease postoperative lung complications such as pleural effusion, atelectasis and pneumonia, we still lack more definitive and clear proofs of inflammatory cytokines encountered during CPB.

**Table:** Abbreviations, acronyms & symbols

ACT	= Activated clotting time
BMI	= Body mass index
C5a	= Complement fragment 5a
CO_2_	= Carbon dioxide
CPAP	= Continuous positive airway pressure
CPB	= Cardiopulmonary bypass
ECG	= Electrocardiogram
ELISA	= Enzyme-linked immunosorbent assay
EtCO_2_	= End-tidal carbon dioxide
FiO_2_	= Fraction of inspired oxygen
ICU	= Intensive care unit
IE	= Inspiration:expiration
IL	= Interleukin
NaHCO_3_	= Sodium bicarbonate
NVG	= Non-ventilation group
NYHA	= New York Heart Association
PEEP	= Positive end-expiratory pressure
ROS	= Reactive oxygen species
SpO_2_	= Oxygen saturation
SPSS	= Statistical Package for the Social Sciences
SRB	= Shanghai Sunred Bio
TNF	= Tumor necrosis factor
TV	= Tidal volume

## INTRODUCTION

Pulmonary complications are major causes of mortality after cardiac surgery^[1]^. The most common pulmonary complications are pleural effusion (up to 95%), atelectasis (88%), prolonged mechanical ventilation (58%), diaphragm dysfunction (54%), and pneumonia (20%)^[2]^. The etiology of these complications in patients undergoing cardiopulmonary bypass (CPB) is still not fully explained. The inflammatory response that occurs during CPB, blood transfusion, hemodilution, aortic cross-clamping, post-sternotomy pain and the associated breathing difficulties, internal mammary artery dissection, hypothermia, and topical cooling are thought to play a role in the development of these complications^[2-4]^.

The mechanism believed to be fundamentally associated with pulmonary complications is the exaggerated inflammatory response that results from artificial perfusion. This response consists of activating the complement system and cytokines as soon as the blood comes into contact with surfaces other than the endothelium. This results in the extravasation of activated leukocytes (activation of lysosomal enzymes) and increased microvascular permeability and pulmonary vascular permeation, thus increasing the risk of developing interstitial edema and atelectasis. Endothelial damage and ischemia/reperfusion result in the production of reactive oxygen species (ROS)^[5]^. Administration of a high fraction of inspired oxygen (FiO_2_) further exacerbates this damage. The mechanical stress resulting from mechanical ventilation itself is reported as another cause of complications^[2,4]^.

Another complicating factor is the cessation of lung ventilation, which aims to prevent blood flow during CPB and allow exploration and an easier operation^[3]^. This application is thought to provide the basis for atelectasis, infection, and effusion during the postoperative period. Therefore, the open lung ventilation approach - which combines a high positive end-expiratory pressure (PEEP) and a high-pressure ventilation - was adopted. However, even though it was reported that these complications decreased in patients undergoing ventilation, it has not yet clinically proven^[1,3]^.

In our study, the effects of low tidal volume ventilation on inflammatory cytokines during CPB were investigated.

## METHODS

### Patient Selection

**Fig. 1:**
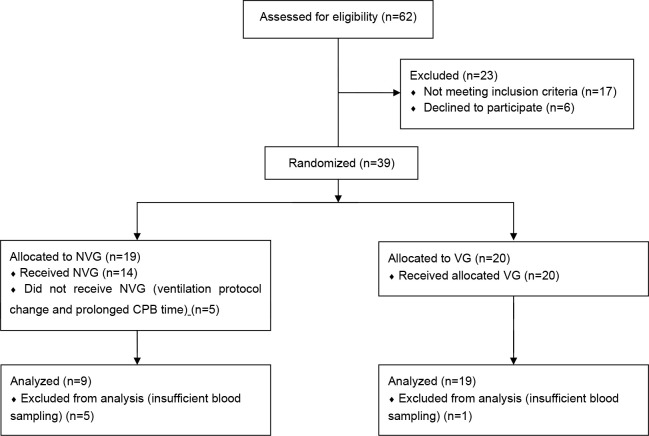
CONSORT flow diagram. CPB=cardiopulmonary bypass; NVG=non-ventilation group; VG=ventilation group

Sixty-two patients admitted to the Harran University Faculty of Medicine Training and Research Hospital between September 2017 and August 2018 and who underwent open-heart surgery were evaluated. Patients over 18 years old and who had open-heart surgery (coronary artery bypass grafting, valve replacement surgery, coronary artery bypass grafting + other cardiac surgeries) were included in the study. The exclusion criteria were: age under 18 years, history of systemic inflammatory diseases, infections, recurrent cardiac surgery, emergency operations, chronic lung disease (chronic obstructive pulmonary disease, lung disease), chronic renal failure, left ventricular dysfunction (left ventricular ejection fraction <50%), congestive heart failure (NYHA class 3-4), overweight (body mass index [BMI] >30), and underweight (BMI <18.5). Seventeen of the participants were excluded according to these criteria. Six patients refused to participate in the study. The remaining 39 patients were included in the study and gave written informed consent. Patients whose ventilation protocol was changed during the operation, patients with prolonged CPB time, and patients whose serum samples were insufficient in analyzes were excluded from the study (Figure 1). Plasma samples of 28 patients were analyzed. This study was approved by the Clinical Ethics Committee (Harran University Scientific Research Project Approval - P0947).

### Surgical Standards, Ventilation Protocols and Subject Groups

Patients who met the inclusion and exclusion criteria were prepared for surgery. Routine monitoring consisted of 5-lead ECG, SpO_2_, and EtCO_2_. After establishing intravenous access, anesthesia was initiated. Midazolam 0.05-0.1 mg/kg IV was administered preoperatively to help control hypertension and tachycardia. After approximately 2 minutes of preoxygenation, anesthesia was induced with 3-10 mg/kg fentanyl, 0.2-0.3 mg/kg etomidate, and 0.6 mg/kg rocuronium. Afterward, patients underwent endotracheal intubation in supine position. Endotracheal tube cuff pressure was kept at 20 cmH_2_O and the position of the tube was checked with lung auscultation. The left radial artery was monitored. Nasopharyngeal and rectal temperature probes were inserted. Anesthesia was maintained with additional doses of fentanyl, rocuronium, and 1-2% sevoflurane in 50/50 oxygen/air mixture. Invasive blood pressure, heart rate, SPO_2_, end-tidal CO_2_, oropharyngeal and rectal temperature, urine output, sevoflurane, air, and oxygen were routinely measured on a gas analysis machine. During the operation, arterial and venous blood gases were monitored.

All subjects underwent standard CPB and all patients were sternotomized. Cannulation was performed with aortic arterial cannulation and unicaval (two-stage)/bicavalvenous cannulation. The priming solution was Ringer's lactate solution 1200 cc, 20% mannitol 100 cc, NaHCO_3_ 20 cc, heparin 1 cc, cefazolin 1 g. Cardiac arrest was achieved with anterograde/retrograde blood cardioplegia, followed by cross-clamping. Mild hypothermia (32-34 °C) was maintained in all operations. Perfusion pressure was maintained at 60-70 mmHg. Full flow was achieved with an arterial flow of 2.4 L/m^2^. During the operation, activated clotting time (ACT) was kept above 480 seconds. Blood oxygenation was controlled with an alpha-stat management approach. After the operation, body temperature was restored by creating a gradient of 10 °C between water and blood until the rectal temperature reached 37.5 °C. CPB was weaned after achieving adequate cardiac performance.

Prior to CPB, all patients were put on volume-controlled ventilation: tidal volume (TV) 6-8 mL/kg, inspiration:expiration (I:E) ratio 1:2, PEEP 5 cm H_2_O, and FiO_2_ 0.5 (targeting SpO_2_ >94%). The respiratory rate was freely adjusted by the anesthesiologist to maintain the end-tidal CO_2_ partial pressure (35 and 45 mmHg). None of the other parameters were allowed to vary.

In the control group (Non-Ventilation Group [NVG], group 1), patients were separated from the ventilator while starting CPB and the lungs were allowed to collapse during CPB without any ventilation. After completion of CPB, ventilation was initiated again with preoperative parameters.

In the Ventilation Group (VG, group 2), ventilation was not stopped while starting CPB. Based on BMI, ventilation was resumed at 15-30% of tidal volume (inspiration:expiration [I:E] ratio of 1:2, PEEP 5 cm H_2_O, FiO_2_ 0.5). Like the control group, ventilation was resumed using preoperative ventilation parameters after CPB. The same parameters were not changed and were also used during CPB.

For postoperative analgesia, all patients were administered tramadol HCl 2 mg/kg without exceeding 400 mg in total. All patients were given antibiotic prophylaxis. Routine treatment was initiated. Patients were extubated when they no longer required inotropes, could respond to commands, had a continuous positive airway pressure (CPAP) of 3-5 cmH₂O, a PaO_2_ >60 mmHg, a PaCO_2_ <45 mmHg, a respiratory rate <20/min, FiO_2_ <0.4, and a chest tube drainage ≤50 mL/h.

### Blood Samples and Examination Procedures

Blood samples were collected before anesthesia induction in the immediate preoperative period (pre-CPB), perioperatively (during CPB, from the coronary sinus), and postoperatively (post-CPB). There are many factors that can affect ILs in the postoperative period. Inflammatory response occurring in surgical incisions and postoperative intensive care follow-up drugs such as corticosteroids can affect these cytokine levels. Therefore, blood samples were collected in the immediate post-CPB period. The collected blood was placed in a container filled with ice and delivered to the laboratory. Afterward, the sterile tube was centrifuged for 5 min at 5,000 rpm. After centrifuging, the supernatant plasma was transferred into Eppendorf tubes and stored at -80 °C until examination. Plasma samples were investigated for TNF-alpha, IL-8, IL-6, and C5a with the human tumor necrosis factor-alpha (Hu-TNF-α) ELISA kit, human complement fragment 5a (C5a) ELISA kit, human interleukin-6 (IL-6) ELISA kit, and human interleukin-8 (IL-8) ELISA kit, according to the manufacturer's instructions (Shanghai Sunred Bio [SRB] Technology Co. Ltd).

### Statistical Analysis

All statistical analyses were calculated by SPSS 22.0 for Windows. The normal distribution was determined by Kolmogorov-Smirnov test and histogram. Non-parametric tests were used for calculations. The continuous variables were expressed as median (min-max). The categorical variables were expressed as numbers and percentages. The differences of continuous variables were calculated by the Mann-Whitney U test for two groups and Kruskall-Wallis test for more than two groups; and the Wilcoxon test was used for repeated measures for two groups and the Wilcoxon signed rank test for more than two groups. Chi-Square test was used to determine the difference between groups of categorical variables. A *P*<0.05 was considered statistically significant. The sizes of the groups were calculated by G*Power statistical software. The least size of each group was calculated as 7 patients with 5% alpha error and 84% power.

## RESULTS

**Fig. 2:**
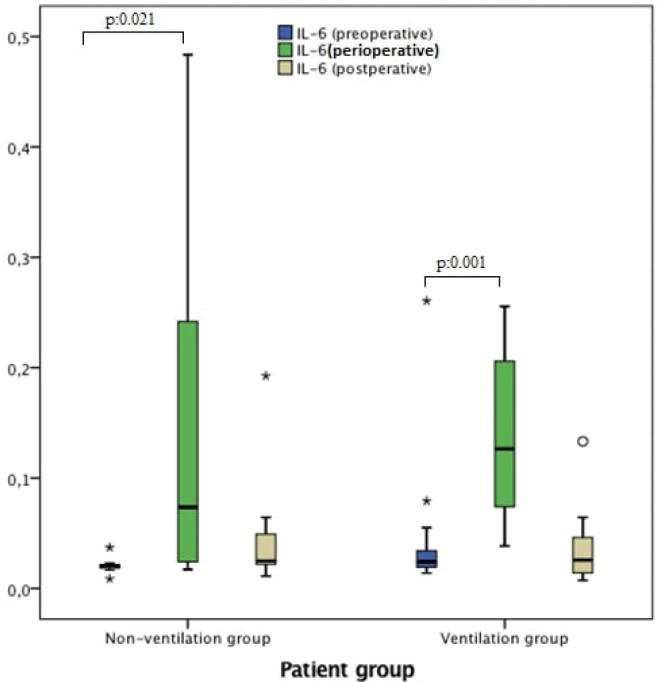
IL-6 measurements in preoperative (pre-CPB), perioperative (during CPB) and postoperative (post-CPB) periods.

**Fig. 3:**
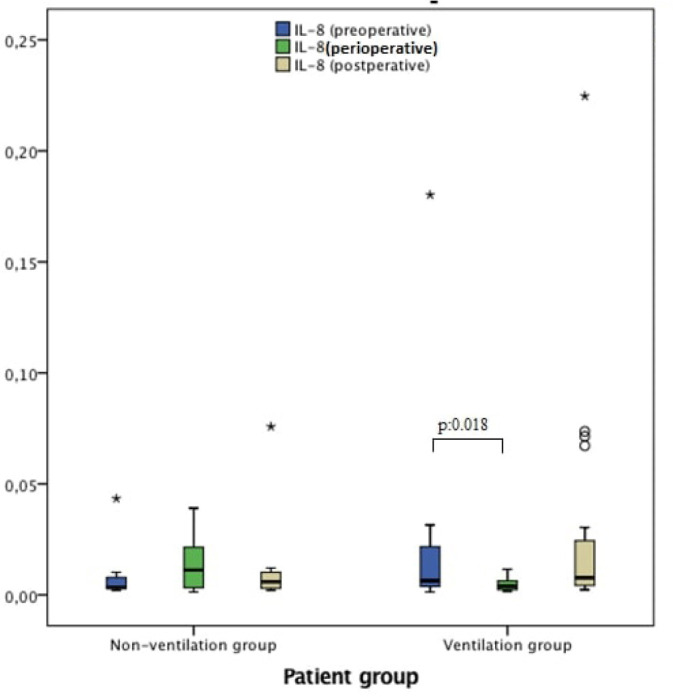
IL-8 measurements in preoperative (pre-CPB), perioperative (during CPB) and postoperative (post-CPB) periods.

**Fig. 4:**
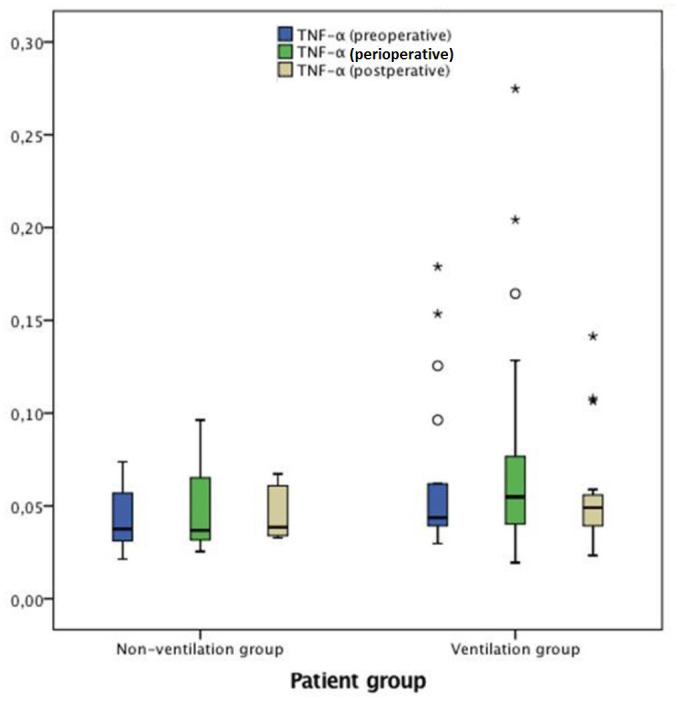
TNF-α measurements in preoperative (pre-CPB), perioperative (during CPB) and postoperative (post-CPB) periods.

**Fig. 5:**
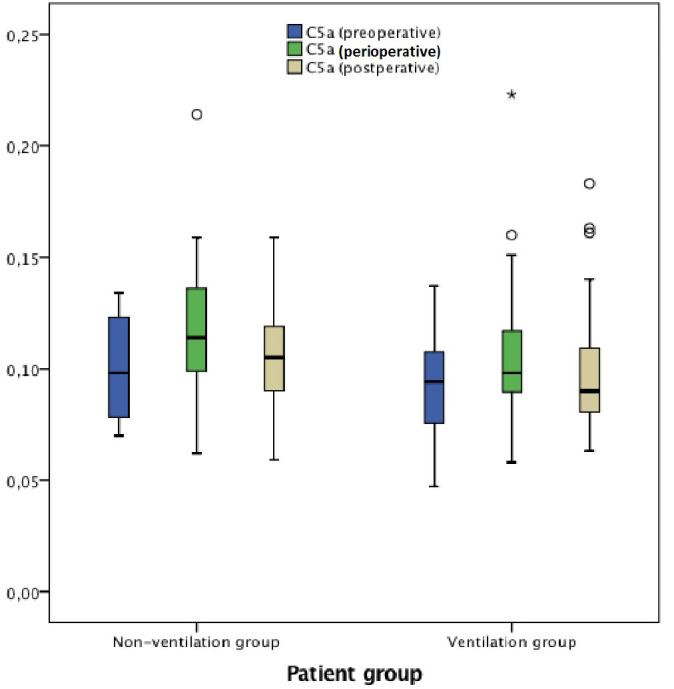
C5a measurements in preoperative (pre-CPB), perioperative (during CPB), and postoperative (post-CPB) periods.

Patient characteristics are summarized in Table 1. Age, total CPB time, cross-clamp time, gender, mortality, extubation time, intensive care unit (ICU) time, hospital length of stay, chronic diseases and operation type were similar in both ventilation and non-ventilation groups (Table 1). The C5a, IL-6, IL-8 and TNF-α measurements and group statistics are shown in Table 2. C5a, IL-6, IL-8 and TNF-α were similar in all measurements in both ventilation and non-ventilation groups (*P*>0.05). IL-6 measurements were significantly higher during CPB compared with before CPB in both non-ventilation and ventilation groups (*P*=0.021 and 0.001, respectively) (Figure 2). IL-8 was significantly lower during CPB compared with before CPB in the ventilation group (*P*=0.018) but there was no difference in the non-ventilation group (*P*=0.314) (Figure 3). C5a and TNF-α were similar in both groups in all measurements (*P*>0.05) (Figures 4 and 5).

**Table 1:** General characteristics of patients.

	Patient group		*P*-value
	Group 1 Non-ventilation group	Group 2 Ventilation group	
	Median (min-max)/n (%)		
Age (years)	52 (24-67)	59 (41-80)	0.110*
Total CPB time (min)	130 (80-220)	130 (86-210)	0.844*
**Cross-clamp time (min)**	72 (60-109)	82 (41-130)	0.694*
Dead	0 (0%)	2 (10.5%)	1**
Alive	9 (100%)	17 (89.5%)	
**Gender**			0.407**
Female	4 (44.4%)	5 (26.3%)	
Male	5 (55.6%)	14 (73.7%)	
Extubating time (min)	420 (210-870)	600 (285-20160)	0.218*
ICU time (days)	2 (1-4)	2 (1-14)	1*
Hospitalization time (days)	11 (7-38)	11 (6-23)	0.961*
**Hypertension**			0.435**
Present	6 (66.7%)	9 (47.4%)	
Absent	3 (33.3%)	10 (52.6%)	
**Diabetes mellitus**			0.630**
Present	1 (11.1%)	5 (26.3%)	
Absent	8 (88.9%)	14 (73.7%)	
**Type of surgery**			0.726**
CABG	8 (88.9%)	15 (78.9%)	
CABG + other surgery	0 (0%)	2 (10.5%)	
Cardiac valve surgery	1 (11.1%)	2 (10.5%)	

* Mann-Whitney U test;

** Chi-square test

# CPB=cardiopulmonary bypass; CABG=coronary artery bypass graft; ICU=intensive care unit

**Table 2:** C5a, IL-6, IL-8 and TNF-α measurements and group statistics.

	Patient group		*P*-value*
	Group 1 Non-ventilation group	Group 2 Ventilation group	
	Median (min-max)		
**C5a (ng/ml)**			
Before CPB (preoperative)	0.1 (0.07-0.13)	0.09 (0.05-0.14)	0.623
CPB (perioperative)	0.11 (0.06-0.21)	0.1 (0.06-0.22)	0.363
After CPB (postoperative)	0.11 (0.06-0.16)	0.09 (0.06-0.18)	0.658
*P*-value** (before CPB *vs*. CPB)	0.441	0.153	
*P*-value** (before *vs*. after CPB)	0.953	0.856	
**IL-6 (ng/ml)**			
Before CPB (preoperative)	0.02 (0.01-0.04)	0.02 (0.01-0.26)	0.201
CPB (perioperative)	0.07 (0.02-2.29)	0.13 (0.04-0.26)	0.787
After CPB (postoperative)	0.03 (0.01-0.19)	0.03 (0.01-0.13)	0.694
*P*-value** (before CPB *vs*. CPB)	0.021	0.001	
*P*-value** (before *vs*. after CPB)	0.260	0.198	
**IL-8 (ng/ml)**			
Before CPB (preoperative)	0 (0-0.04)	0.01 (0-0.18)	0.389
CPB (perioperative)	0.01 (0-0.04)	0 (0-0.01)	0.279
After CPB (postoperative)	0.01 (0-0.6)	0.01 (0-0.22)	0.658
*P*-value** (before CPB *vs*. CPB)	0.314	0.018	
*P*-value** (before *vs*. after CPB)	0.208	0.398	
**TNF-α (ng/ml)**			
Before CPB (preoperative)	0.04 (0.02-0.07)	0.05 (0.03-0.6)	0.218
CPB (perioperative)	0.04 (0.03-2.46)	0.05 (0.02-0.27)	0.476
After CPB (postoperative)	0.04 (003-2.53)	0.05 (0.02-1.22)	0.571
*P*-value** (before CPB *vs*. CPB)	0.515	0.601	
*P*-value** (before *vs*. after CPB)	0.374	0.520	

* Mann-Whitney-U test;

** Wilcoxon test

## DISCUSSION

The suspension of ventilation during CPB is the common practice for respiratory management^[1]^. After cross-clamping, the lungs are isolated from systemic circulation during CPB, when the bronchial arteries provide blood flow to the lungs. This flow is considerably small compared to that of the pulmonary artery (approximately 3% to 5%). The increased interstitial permeability and the reduced perfusion of the lungs that result from CPB initiate the inflammatory response via cytokines. After the procedure, this inflammatory response exacerbates with reperfusion, and the oxygen radicals produced trigger lung damage and create ground for postoperative complications. Atelectasis and interstitial fluid collection during the re-ventilation of lungs produce a ventilation-perfusion mismatch, further aggravating this process^[2,6]^. It is suggested that maintaining lung ventilation may be beneficial in preventing these mechanisms that develop during CPB. This ventilation will maintain pulmonary blood flow, which will presumably reduce inflammation and prevent ischemia-reperfusion damage. This technique is also supposed to maintain pulmonary mechanics and reduce postoperative complications^[7]^.

In this study, we examined the serum C5a, IL-6, IL-8 and TNF-α levels of CPB patients who did and did not undergo ventilation under certain criteria. When the groups with and without ventilation are compared, there is no difference in cytokine levels. However, when the groups were handled individually, significant differences were observed in the levels of IL-6 and IL-8 during CPB. The fact that IL-6 increased in both groups reminds us once again that blood contact with non-endothelial structures strongly promotes inflammatory processes. A similar increase was also observed in IL-8 levels in the non-ventilation group; however, this finding was not statistically significant (*P*=0.314). In the ventilation group, IL-8 levels significantly decreased during CPB (*P*=0.018). This finding is important because it demonstrates that low tidal volume ventilation can reduce the inflammatory response. Besides, IL-6 and IL-8 describe different aspects of inflammation. Unlike IL-6, IL-8 is a chemokine that can also be produced from airway smooth muscle cells^[8]^. Therefore, low IL-8 levels should be expected in the anti-inflammatory response that occurs in the lungs. Also, IL-6 functions as both a pro-inflammatory cytokine and an anti-inflammatory myokine. The role of IL-6 as an anti-inflammatory myokine is mediated by its inhibitory effects on TNF-alpha and IL-1 and activation of IL-1Ra and IL-10^[9]^. Therefore, the decrease in IL-8 and the increase in IL-6 levels can also be interpreted as an indicator of an anti-inflammatory response.

Although studies have demonstrated that atelectasis and infection are reduced with low tidal volume ventilation^[10,11]^, we did not find such a difference in our ventilation and non-ventilation groups. Similar to our study, Fiorentino et al.^[12]^ did not find any difference between the TNF-α, IL-1β, IL-18, IL-6, IP-10, CXCL-8 and IL-10 levels of patients that did and did not receive ventilation support. An animal study supported these findings by reporting that IL-6, IL-8 and IL-10 levels were similar in all subject groups^[7]^. Contrary to these two studies, Gaudriot et al.^[13]^ reported that IL-10 and proinflammatory TNF-α levels of patients on ventilation were reduced. This was ascribed to the inability of low volume ventilation to evenly aerate every region of the lungs, resulting in relative ischemia-reperfusion damage. In addition, ventilator damage and hyperoxia resulting from high FiO_2_ exacerbate this damage at least as much as non-ventilation^[14]^. Studies have examined multiple different methods to achieve lung ventilation and reported considerably different results. Oxygenation was shown to increase in patients undergoing CPAP treatment but there is no evidence confirming this effect was permanent^[15]^. It has been demonstrated that ventilation with PEEP in heart surgery prevents atelectasis^[16]^, reduces inflammation^[10]^, and improves pulmonary mechanics. However, it is not yet clear whether it is clinically beneficial^[11]^. It was reported that hyperoxia and lung ventilation had minimal effect on postoperative organ dysfunction, length of hospital stay, and heart surgery mortality outcomes^[17]^.

Another point of discussion is the increased lymphatic flow resulting from increased blood flow associated with ventilation. Normally, lung lymph flow is markedly reduced by the cessation of ventilation during CPB and the decreased blood flow in the bronchial arteries following cross-clamping^[18]^. It is expected that allowing the lungs to function with low tidal volume will result in increased bronchial arterial flow, and subsequently, increased lymphatic flow^[14]^. This will reduce pulmonary edema. However, animal studies suggest that high oxygen concentrations associated with ventilation or limited lung capacity resulting from conditions such as lobectomy/pneumonectomy may modulate pulmonary edema after reperfusion injury^[19]^. The fact that our study groups were not significantly different can be interpreted as that this increase in lymphatic flow is partial or insufficient. Of course, this conclusion is inferential and it is clear that further studies are needed to better understand the lymphatics of the lungs.

## CONCLUSION

Ventilation management during cardiothoracic surgery is intricate and the correct management of intraoperative ventilation during CPB is still an unsettled issue in the guidelines. According to our findings, low tidal volume ventilation on CPB may have a protective role against exaggerated inflammatory response in lungs that result from artificial perfusion. Future studies on this subject will increase our knowledge.
